# Assessment of Mean Platelet Volume in Patients with Systemic Lupus Erythematosus

**DOI:** 10.2174/1874312901812010129

**Published:** 2018-08-31

**Authors:** Lisandra Torres Hartmann, Ana Paula Alegretti, Alice Beatriz Mombach Pinheiro Machado, Eduardo Ferreira Martins, Rafael Mendonça da Silva Chakr, Andrese Aline Gasparin, Odirlei André Monticielo

**Affiliations:** 1 Department of Clinical Pathology, Hospital de Clínicas de Porto Alegre, Porto Alegre, Brasil; 2 Division of Rheumatology, Department of Internal Medicine, Hospital de Clínicas de Porto Alegre, Universidade Federal do Rio Grande do Sul, Porto Alegre, Brasil

**Keywords:** Mean platelet volume, Systemic lupus erythematosus, Systemic lupus erythematosus disease activity index, Autoimmunity, Biomarker, ESR

## Abstract

**Introduction:**

The Mean Platelet Volume (MPV) is a platelet activation biomarker that has been recently correlated with disease activity in SLE. We aimed to evaluate the MPV in patients with SLE comparing it with healthy individuals, to study the correlation between MPV and SLE Disease Activity Index (SLEDAI) in SLE patients and to analyze possible correlation between MPV and Erythrocyte Sedimentation Rate (ESR), C-Reactive Protein (CRP), and complement components C3 and C4.

**Methods:**

This is a cross-sectional study in which 81 patients with SLE according to the American College of Rheumatology (ACR) diagnostic classification criteria and 58 healthy controls were included. Active disease was defined as SLEDAI>0.

**Results:**

Patients with active SLE had decreased MPV when compared to inactive disease group (10.0±0.7fL *vs*. 10.7±1.0fL, *p*=0.005, respectively) and when compared to control group (10.9±1.0fL, *p*<0.001). Our study found a weak negative correlation between the SLEDAI and the MPV (r=-0.29, *p*=0.009). There was no correlation between MPV and CRP, ESR, C3 and C4. Also, no correlation between SLEDAI and CRP, ESR, C3 and C4 was found.

**Conclusion:**

MPV decreases in patients with active SLE and is inversely correlated with SLEDAI.

## 1 INTRODUCTION

Systemic Lupus Erythematosus (SLE) is a chronic autoimmune inflammatory disease, with a pleomorphic nature regarding the pathogenesis and onset of clinical manifestations. The development of autoimmunity in SLE is related to the loss of immunological tolerance and immunoregulatory control [1].

SLE is a disease which evolves with periods of activity and remission. It is essential for the clinician, during the patient's follow-up, to verify in which phase the same is found [1]. The SLE activity can pdf-space/>be measured by SLEDAI (Systemic Lupus Erythematosus Disease Activity Index), which is a complex tool composed of 24 clinical and laboratory variables, and it requires training and knowledge for its application [2]. Measurement of serum levels of individual complement components is commonly used to diagnose and assess disease activity in SLE. Significantly decreased values of serum C1q, C3 and C4 have been associated with increased SLE disease activity manifested by active nephritis and extrarenal involvement. Besides that, the persistence of C3 low levels after treatment is an indicator of worse outcome [3, 4]. Nevertheless, there is still no reliable laboratory test that can independently quantify this disease activity [5, 6]. The discovery of new biomarkers capable of monitoring the activity of the disease in a more practical way is essential.

The Mean Platelet Volume (MPV) is a parameter detected by hematological analyzers during routine blood count. The MPV was shown to be a reliable inflammatory marker in several diseases such as ulcerative colitis [7], ankylosing spondylitis, rheumatoid arthritis, rheumatic fever, and even chronic obstructive pulmonary disease [8-10], besides being associated with a high risk of stroke [11] and myocardial infarction [12]. This index has been shown to be associated with the inflammatory process, and it is also an important marker of platelet activation and function [5, 6, 13-18]. Several studies suggest that MPV may be a good marker of disease activity in SLE, presenting low cost and wide availability [5, 13, 15, 19]. Recent studies have found that MPV significantly reduced in patients with active disease [15, 19], including negative correlation with SLEDAI [19]. Other study performed in a juvenile SLE (JSLE) population, however, showed significantly higher MPV values in patients compared to healthy controls and in the active disease group when compared to the remission group. There are still doubts regarding the possibility of using MPV as a disease activity biomarker in adult patients with SLE.

Our study aimed to evaluate MPV in adult patients with SLE, and to compare MPV values among SLE patients and healthy individuals. Moreover, we studied the correlation between MPV and disease activity index (SLEDAI) in SLE patients and analyzed a possible correlation among MPV and Erythrocyte Sedimentation Rate (ESR), C-Reactive Protein (CRP), and complement components C3 and C4.

## 2 MATERIALS AND METHODS

### 2.1 Patients and Study Design

This is a cross-sectional study with the inclusion of 139 participants. The study population consisted of 81 SLE patients who were followed up at the outpatient clinic of the Rheumatology Service of the Hospital de Clínicas de Porto Alegre (HCPA). Patients were selected consecutively between October 2015 and July 2016. The diagnosis of the disease was established based on the presence of at least four of the 11 criteria of diagnostic classification criteria proposed by the American College of Rheumatology (ACR) in 1982 and revised in 1997 [20]. Patients with an overlap of other diffuse connective tissue diseases (except Sjögren's syndrome and antiphospholipid antibody syndrome) and non-SLE-related hematological diseases such as thalassemia, myeloproliferative disorders, myelofibrosis, Bernard-Soulier syndrome, and anomaly of May-Hegglin were excluded. Patients with previous or current history of neoplasm, diabetes, uncontrolled systemic arterial hypertension, heart disease, hyperthyroidism, splenectomy, thrombocytopenia, inflammatory bowel disease, psoriasis, acute and chronic infections (hepatitis B, hepatitis C, human immunodeficiency virus infection, tuberculosis and syphilis) were also excluded, as well as patients who underwent blood transfusions three months prior to screening. Healthy individuals were selected among volunteers from the HCPA blood bank, matched by sex and ethnicity. The research project was approved by the ethics committee of the HCPA and all participants were included only after reading, understanding and signing the informed consent form (ICF) in accordance with the Helsinki Declaration.

### 2.2 Clinical and Laboratory Variables

At the time of the consultation, disease activity index was calculated through SLEDAI and disease chronicity index through SLICC damage index [21], respectively. Patients with SLEDAI> 0 were considered to have active SLE. Data were also collected regarding the treatment used at the time of the consultation and detection of possible exclusion criteria. The patient's chart was reviewed to confirm the diagnostic classification criteria. Blood collection should have been performed within 10 days prior to consultation, when the patient was invited to participate in the study and signed the ICF.

### 2.3 Analytical Methods Used

Blood counts and platelets were measured on the Sysmex XE 5000 automation equipment. A total of 5 ml of venous blood was taken in an EDTA tube from every participant. All samples were analyzed within 1 hour after collection. The platelets were analyzed by impedance methodology, and the MPV index was calculated by the equipment when the platelet test was performed. MPV was obtained by dividing the platelet count (called platelet hematocrit or platelet volume ratio, weighted for platelet frequency) by the number of platelets.

The C3 and C4 and CRP (C-Reactive Protein) exams were measured by immunoturbidimetry in the SIEMENS-ADVIA1800 automation equipment. C1q levels measurement is not a part of our outpatient routine exam request and could not be assessed. Anti-dsDNA was performed by immunofluorescence, and anticardiolipin antibodies, performed by the Liaison automation equipment. ESR was performed on the Alere Roller 20 equipment. Urinary sediment was performed on the IQ200-IRIS automation equipment. The lupus anticoagulant was analyzed in the BCS-Siemens automation equipment using reagent LA1 and LA2 (both produced with Russel viper venom at different concentrations) with LA1 screening and LA2 being confirmatory.

### 2.4 Statistical Analysis

Data analysis was performed in SPSS 16.1 software. The comparison of the two groups (patients and controls) was determined by Student's t-test. The results were presented as means and standard deviations. The correlation between the variables was calculated by Pearson's and Spearman's coefficients. ROC curve was performed to determine the value of the MPV Cutoff as a disease activity biomarker. The sensitivity, specificity, positive predictive value and negative predictive value, likelihood ratio and confidence interval and the p-value were also calculated.

To detect a difference between the MVP outcome of SLE patients and the healthy controls, the results obtained by Yavuz S., *et al.* found a standard deviation of 2.7 and 0.52 respectively. With a ratio of 3 cases to 2 controls, considering a power of 80% and significance level of 5%, expecting a difference of 1 fL between the groups, a ‘n’ (sample) of 90 patients and 60 controls will be necessary [5].

## 3 RESULT

This study consisted of 81 patients with SLE and 58 healthy controls. The patients were 96.4% female, 72.3% Euro-descendants, mean age 42.7±12.3 years and mean disease duration of 12.8±7.8 years. Fifty-four (54.4%) patients had active disease at the time of evaluation. The comparison of clinical and laboratory characteristics of patients with inactive and active disease is described in Tables **1** and **2**. The group of healthy controls consisted of 89.7% of women, 89.7% Euro-descendants with a mean age of 36.4±11.9 years. Except for age, total leukocytes count and hemoglobin, SLE and control groups were similar.

The mean MPV of SLE patients was 10.3±0.9 fL, while that of the control group being 10.9±1.0 fL (*p*<0,001). When we analyzed patients with SLE, the group with active disease presented mean MPV of 10.0±0.7fL and the group with disease in remission 10.7±1.0 fL (*p* <0.001).

Table **3** describes the treatment received by the patients. There was not a significant difference of how the patients were treated when comparing the SLE patients with active or inactive disease.

The MPV showed a negative correlation with the SLEDAI, which was, although weak, statistically significant (*r*=-0.29, *p*=0.009) Fig. (**1**). MPV results did not show statistically significant correlation with CPR, ESR and C3 and C4 complements (*p*=0.562, *p*=0.796, *p*=0.427, *p*=0.977, respectively). SLEDAI also did not present a statistically significant correlation with CRP, ESR, C3 and C4 (*p*=0.856, *p*=0.640, *p*=0.300, *p*=0.415, respectively). Fig. (**2**) shows the sensitivity versus the specificity for different cutoff levels of MPV, ESR, CRP, C3 and C4.

## 4 DISCUSSION

Our study found significantly lower values of MPV in patients with active SLE when compared to patients in remission. In SLE, lower platelet size has been linked to platelet activation. Platelet system activation is a key event in the pathogenesis of SLE. Immune Complexes (ICs) from SLE sera are potent activators of platelets through their binding to FcγRIIA (CD32) on platelets’ surface. SLE ICs could also act through Toll-Like Receptor-4 (TLR-4) or TLR-7, as TLR-4 and TLR-7 agonists promote platelet activation [22, 23]. Antiphospholipid antibodies (ApL) can mediate platelet activation directly through interaction with a platelet’s plasma membrane, by binding diverse platelet receptors and/or by promoting complement deposition on platelets [24]. Lastly, infectious agents such as a virus can activate platelets in SLE. Upon viral infection, platelets are activated through TLR7, which induces a change in their phenotype, leading to the formation of platelet-neutrophil aggregates. These aggregates ultimately lead to platelet internalization and thrombocytopenia without the promotion of thrombosis [25]. Upon activation, platelets promote type I interferon production, NETosis, dendritic cell activation, and T and B lymphocyte activation, all essential events contributing to the development of SLE [26].

Other studies have found results similar to ours [13, 15, 19]. In rheumatoid arthritis, there is a study that also demonstrated diminished results of MPV in the active disease. In this study, after the patients' treatment, the values of the MPV increased [27]. Similar results were obtained in a study with adult patients with active ankylosing spondylitis, where MPV was analyzed before and after treatment, and the MPV values showed a significant increase after the treatment [16].

MPV has been considered a possible marker of platelet activation and inflammatory process [13]. The pathophysiology of SLE involves the presence of inflammatory cytokines and deregulation of the complement system, which interferes with the activation of platelets [15]. A probable mechanism that may explain the relationship between reduced MPV and disease activity is the consumption of large platelets at sites of inflammation [6, 15].

Although our MPV results did not show statistically significant correlation with the parameters of CRP, ESR and C3 and C4 complement components, we found a negative correlation between MPV and SLEDAI, similar to that found by Khan A., *et al.* [19]. Although the correlation was weak, it confirms that the diminished results of MPV in patients with active SLE are linked to the disease activity. It is well known that CRP and ESR are not good biomarkers of disease activity in SLE. However, as C3 and C4 complement components are 1 part of the 24 items evaluated in SLEDAI, it would be expected to have some correlation with the variation in disease activity, a fact that was not observed in the present study. A plausible explanation would be a bias selection of patients with low complement intake as one of the scoring items in SLEDAI.

Yavuz S.,* et al.*, in a study with individuals with juvenile SLE, found higher MPV values in patients with active disease compared to the remission group [5]. In the same study, there was also a positive correlation with SLEDAI, ESR, CRP and protein and creatinine ratio in a urinary sample [5]. Perhaps this discrepancy occurs due to the inherent characteristics of the treatment or study population. SLE is a chronic autoimmune disease that intersperses periods of activity and remission, and the JSLE presents a worse prognosis than SLE in adults, requiring a more aggressive immunosuppressive therapy. Another aspect that should be considered is that the study with JSLE was conducted on a small sample of patients, having evaluated only 20 children. Other previous studies have shown a probable relationship between high values of MPV and active inflammatory disease [6, 28]. Recently another study with 128 patients found higher MPV values in active LES and a moderate correlation between MPV and SLEDAI score [29]. Several factors may explain these differences: the pre-analytical handling of the samples, which is a key issue; the presence of antiphospholipid antibodies, and several other known or unknown cofactors, making difficult the clinical assumptions based on MPV [23, 30].

Our study also showed decreased MPV results in active patients when compared to healthy controls, this result was consistent with the study performed in patients with inflammatory bowel disease; however, in our study, the results of the patients in remission were similar to the results of the MPV in the controls. And in the study performed with inflammatory bowel disease, the results of the MPV in patients in remission were also decreased when compared to control group [31]. In a Meta-analysis evaluating the relationship between hematological indices and autoimmune rheumatic diseases, Hao and coworkers did not find significative difference related to MVP comparing SLE patients with healthy control group. However, in relation to this analysis, only two studies were included and there was important heterogeneity between them [32].

The MPV is an easily measurable parameter, and has shown high sensitivity (86%) in a previous study so it may be considered a potential candidate for activity biomarker in SLE [15]. Although the MPV presented pre-analytical interferences related to the time of collection, sample storage and the anticoagulant used [28], these variables were controlled in our study. In addition, we evaluated the treatment used in the patients of the active and inactive groups, and there was no statistically significant difference between them. Our results were similar to the recently performed study with patients with active and inactive SLE at similar sampling [15].

Total leukocyte count and hemoglobin were reduced in active SLE Table **1**. No difference was found between active and inactive SLE regarding the medications used by the patients Table **2**. These results are consistent with those from the literature.

Our study has several limitations. It cannot establish a causal relationship because of its transversal design. There was no age pairing between the SLE groups and the control group. The active disease group had an average SLEDAI of 4 (disease with mild activity), while the other studies included patients with more active disease in this group (SLEDAI mean> 16) [5, 19]. Even though SLEDAI showed little difference between the active SLE and remission groups, there was no difference in treatment between these groups. Patients with antiphospholipid antibody syndrome were not excluded from this study. The increase of MPV in patients with this syndrome is described in comparison to control patients [33]. However, only 1 patient in the SLE group in remission and 1 patient in the active SLE group presented APS. Previous studies have shown increased MPV in patients with Chronic Obstructive Pulmonary Disease (COPD) [9, 34, 35]. In our study 5 patients with COPD were included, 4 in the active SLE group and 1 in the SLE remission group. This bias was conservative, because despite presenting a greater number of patients with COPD, MPV was still significantly lower in the active SLE group. There was a difference between the use of aspirin between the groups (10 patients with active SLE and 7 patients with SLE in remission), but previous studies did not find a significant effect on the MPV with the use of low dose aspirin (up to 100 mg daily) [36, 37]. Another relevant point is the impossibility of defining how the MPV could be used against other biomarkers of systemic inflammation (CRP and ESR), since the ROC curves were similar.

Although the number of patients included in the study was slightly lower than the sample size, there was a statistically significant difference of the MPV in the active SLE group in relation to inactive SLE and controls.

The mean MPV difference between the groups, however, was small (less than 1 fL). The least variation in the MPV values observed in our study (SD ± 0.7, ± 1.0 and ± 1.0 in the active SLE, SLE in remission and controls, respectively) compared to those found by Yavuz *et al* (SD ± 2.7 ± 0.86 and ± 0.52 in the active LES, SLE in remission and controls, respectively) may explain this statistical significance, even with a lower MPV difference. However, the clinical relevance of this small difference is quite questionable.

At last, the platelet-derived microparticles (PMPs) were not evaluated in our work. It was recently shown that PMPs are generated after platelet activation and were shown to play a role in hemostasis, thrombosis, cancer, inflammation and autoimmunity. Moreover, circulating PMPs were increased in lupus nephritis and correlated with high blood pressure and proteinuria, two prognostic factors in lupus suggesting a direct role of PMPs in the pathogenesis of lupus nephritis [26, 38].

We conclude that MPV is reduced in patients with active SLE and presents an inverse correlation with SLEDAI. Despite the difference between MVP values and between active and inactive SLE patients, the results may not be clinically relevant. Prospective longitudinal studies are needed to better characterize the fluctuation of MPV in different stages of disease activity to more clearly define the role of MPV in SLE.

## Figures and Tables

**Fig. (1):**
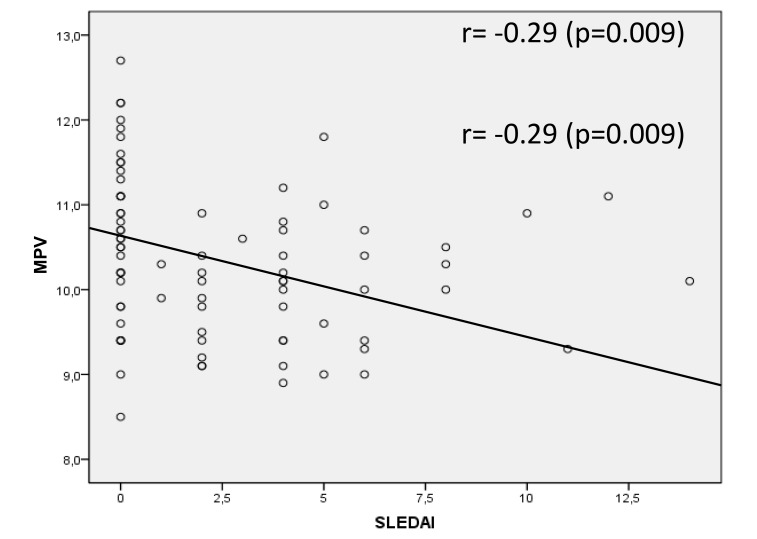
The correlation between MPV and SLEDAI.

**Fig. (2):**
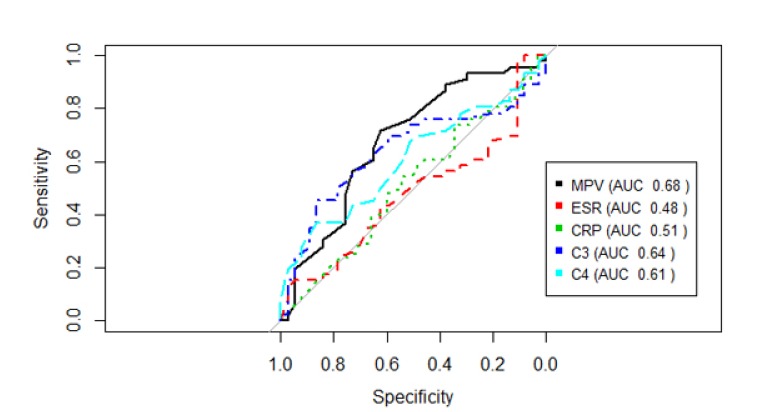
Sensitivity versus the specificity for different cutoff levels of MPV, ESR, CRP, C3 and C4.

**Table 1:** Demographic, clinical, and laboratorial features of SLE patients.

Patient’s Features	Whole (n=81)	SLEDAI=0 (n=37)	SLEDAI>0 (n=44)	*p* Value*	Controls (n=58)
Age (years±SD)	42.7±12.3	43.6±12.3	41.8±12.3	0.519	36.4±11.9^a^
Disease duration (years±SD)	12.8±7.8	13.6 ±8,3	12.1±7.3	0.425	-
Malar rash (%)	50.7	42.4	57.5	0.243	-
Discoid rash (%)	8.2	12.1	5.0	0.400	-
Photosensitivity (%)	68.5	63.6	72.5	0.456	-
Oral ulcers (%)	41.1	27.3	52.5	**0.034**	**-**
Arthritis (%)	72.6	69.7	75.0	0.793	-
Serositis (%)	15.3	12.1	17.9	0.533	-
Nephritis (%)	47.9	42.4	52.5	0.482	-
Neurologic disorders (%)	13.7	18.2	10.0	0.496	-
Hematologic disorders (%)	71.2	63.6	77.5	0.207	-
Hemolytic anemia (%)	30.1	21.2	37.5	0.200	-
Leukopenia/ Lymphopenia (%)	21.5	10.8	31.0	0.053	-
Thrombocytopenia (%)	2.5	-	4.5	0.498	-
Immunologic disorders (%)	65.7	59.4	71.1	0.325	-
Anti-dsDNA (%)	12.8	2.8	21.4	**0.017**	**-**
Anti-Sm (%)	18.4	10.5	26.3	0.405	-
Anticardiolipin (%)	17.1	18.8	15.8	0.761	-
Lupus anticoagulant (%)	5.4	8.6	2.6	0.339	-
False positive VDRL (%)	1.5	0.0	2.6	1.000	-
ANA (%)	98.6	100.0	97.6	1.000	-
Anti-Ro/SSA (%)	33.8	21.2	44.7	**0.046**	**-**
Anti-La/SSB (%)	12.7	3.0	21.1	**0.033**	**-**
Anti-RNP (%)	26.4	18.2	33.3	0.185	-
Sjögren's syndrome (%)	1.5	0.0	2.9	1.000	-
Antiphospholipid syndrome (%)	2.9	3.0	2.7	1.000	-
SLEDAI^c^	2 (0-4)	0(0-0)	4 (2-6)	**<0.001**	**-**
SLICC damage index^c^	0 (0-1)	0(0-1)	1(0-2)	0.304	-
MPV (fL±SD)	10.3±0.9	10.7±1.0	10.0±0.7	**0.001**	10.9±1.0^a^
ESR (mm/h±SD)	29.3±17.7	29.0±18.9	29.5±16.8	0.896	-
CRP (mg/dL)^c^	2.8(1.4-5.3)	2.9(1.3-5.3)	2.6(1.5-5.2)	0.949	-
C3 (mg/dL±SD)	105.3±27.1	110.1±21.0	101.3±30.9	0.135	-
C4 (mg/dL±SD)	19.6±10.0	20.9±8.8	18.6±10.1	0.294	-
Euro-derived ethnicity (%)	-	62.2^a^	79.5	-	89.7^a^
Female (%)	-	97.3	95.5	-	89.7
WBC (103/mm3)	-	6.55±2.4^b^	5.95±3.1^a,b^	-	7.47±1.9
Hemoglobin (g/dl)	-	12.7±1.0^a^	12.1±1.4^a^	-	14.1±0.9^a^
Platelets (103/mm3)	-	244.0±49.5	270.4±105.7	-	274.6±57.8

Abbreviations: ANA: Antinuclear Antibody; CRP: C-Reactive Protein; ESR: Erythrocyte Sedimentation Rate; SD: Standard Deviation; SLEDAI: Systemic Lupus Erythematous Disease Activity Index; SLICC: Systemic Lupus International Collaborating Clinics; MPV: Mean Platelet Volume; VDRL: Venereal Disease Research Laboratory Test; WBC: White Blood Cells. *Chi square test for qualitative variables and ANOVA for quantitative variables, and Tukey test. ** Chi square test for qualitative variables and Mann-Whitney for asymmetric quantitative variables or Student’s *t* test for symmetric quantitative variables. ^a^ *p*-value<0.05 between patients and controls. ^b^ *p*-value<0.05 between active and inactive patients. ^c^ Median (interquartile range).

**Table 2:** Description of treatment in patients with inactive and active lupus.

Medications	SLEDAI=0 (n=37)	SLEDAI>0 (n=44)	*p* Value*
Corticosteroids (%)	32.4	47.8	0.183
Immunosuppressive dose (%)	37.8	23.9	0.229
Antimalarial (%)	86.5	73.9	0.182
Azathioprine (%)	40.5	45.7	0.663
Cyclophosphamide (%)	5.4	13.0	0.289
Micophenolate (%)	11.1	11.4	1.000
Methotrexate (%)	8.1	4.3	0.652
Anti-hypertensive drugs (%)	45.9	63.0	0.128
Statins (%)	18.8	24.3	0.771

ANA: Antinuclear Antibody; CRP: C-Reactive Protein; ESR: Erythrocyte Sedimentation Rate; SD: Standard Deviation; SLEDAI: Systemic Lupus Erythematous Disease Activity Index; SLICC: Systemic Lupus International Collaborating Clinics; MPV: Mean Platelet Volume; VDRL: Venereal Disease Research Laboratory Test. *Chi square test for qualitative

**Table 3:** Laboratory parameters for identification of SLEDAI> 0.

-	MPV	CRP	ESR	C3	C4
Sensitivity	61%	41%	41%	23%	54%
Specificity	24%	63%	78%	81%	35%
PPV	49%	58%	69%	59%	50%
PNV	35%	46%	53%	47%	40%
Cutoff level	9.85fL	3.65mg/dL	35.5mm/Hg	122.5mg/dL	15.5mg/dL

PPV: Positive Predictive Value; PNV: Negative Predictive Value.
